# Asymmetrical Obstacles Enable Unilateral Inertial Focusing and Separation in Sinusoidal Microchannel

**DOI:** 10.34133/cbsystems.0036

**Published:** 2023-06-19

**Authors:** Haotian Cha, Yuchen Dai, Helena H. W. B. Hansen, Lingxi Ouyang, Xiangxun Chen, Xiaoyue Kang, Hongjie An, Hang Thu Ta, Nam-Trung Nguyen, Jun Zhang

**Affiliations:** ^1^ Queensland Micro- and Nanotechnology Centre, Griffith University, Nathan, Queensland 4111, Australia.; ^2^ School of Engineering, University of Tasmania, Churchill Avenue, Tasmania 7005, Australia.; ^3^ Bioscience Discipline, School of Environment and Science, Griffith University, Nathan, Queensland 4111, Australia.

## Abstract

Inertial microfluidics uses the intrinsic fluid inertia in confined channels to manipulate the particles and cells in a simple, high-throughput, and precise manner. Inertial focusing in a straight channel results in several equilibrium positions within the cross sections. Introducing channel curvature and adjusting the cross-sectional aspect ratio and shape can modify inertial focusing positions and can reduce the number of equilibrium positions. In this work, we introduce an innovative way to adjust the inertial focusing and reduce equilibrium positions by embedding asymmetrical obstacle microstructures. We demonstrated that asymmetrical concave obstacles could break the symmetry of original inertial focusing positions, resulting in unilateral focusing. In addition, we characterized the influence of obstacle size and 3 asymmetrical obstacle patterns on unilateral inertial focusing. Finally, we applied differential unilateral focusing on the separation of 10- and 15-μm particles and isolation of brain cancer cells (U87MG) from white blood cells (WBCs), respectively. The results indicated an excellent cancer cell recovery of 96.4% and WBC rejection ratio of 98.81%. After single processing, the purity of the cancer cells was dramatically enhanced from 1.01% to 90.13%, with an 89.24-fold enrichment. We believe that embedding asymmetric concave micro-obstacles is a new strategy to achieve unilateral inertial focusing and separation in curved channels.

## Introduction

Cell manipulation and separation are indispensable in biological research, disease diagnosis, cell therapy, drug screening, and genetic analysis [1–6]. Microfluidic technologies that manipulate and control fluid flow at the microscale are emerging for cell manipulation and separation [7–11]. Currently, reported microfluidic technologies for cell manipulation and separation can be classified as either active or passive methods based on the sources of manipulating forces. In general, active methods apply external electrical [12–14], magnetic [15–17], acoustic [18,19], optical [20,21], and thermal force fields [22,23]. Active separation technologies offer the benefits of precise manipulation and real-time control by simply adjusting the external force fields. However, they are limited by relatively low-throughput and complex external supportive equipment. In contrast to active technologies, passive technologies rely on intrinsic channel geometry or fluid dynamics, eliminating the requirement for complex equipment. Typical passive methods include microfilters [24,25], pinched flow fractionation [26,27], deterministic lateral displacement [28–30], inertial microfluidics [31–33], viscoelastic microfluidics [34–36], and diffusiophoresis [37]. Passive methods possess the advantages of high throughput, easy operation, and high efficiency.

Among the above passive technologies, inertial microfluidics has attracted a broad interest because of its high throughput, low cost, simple structure, and precise manipulation [38]. In Newtonian fluids within finite Reynolds number flows, the dispersed particles migrate across the streamlines to several equilibrium positions in a straight channel depending on the synergistic effect between 2 inertial lift forces: shear gradient lift force and wall lift force [38–40]. The balance of the 2 forces determines the numbers and locations of particle focusing positions within the channel cross sections. For example, particles typically focus on 4 equilibrium positions facing the center of each wall in a square straight channel [41]. However, focused particles are dispersed at 4 equilibrium positions within the channel cross section, bringing challenges for particle detection and separation. To modify and reduce focusing number and positions, several strategies have been proposed, such as adjusting the aspect ratio [42] and shape [43] of channel cross sections. For instance, in a straight channel with a square cross section, particles tend to concentrate at 4 equilibrium positions located near the center of each channel wall. However, in a straight channel with a low aspect ratio (height/width) ranging from one-third to one-half, these 4 equilibrium positions can decrease to 2, where particles become focused near the center of the top and bottom walls. Furthermore, when a series of constrictions in height are introduced, the focusing positions are further reduced to a single position [44]. Additionally, another group altered the cross-sectional shape of the channel from rectangular to triangular or semicircular and found that, near the middle of the channel, 3 focusing positions were observed [43].

Furthermore, the introduction of channel curvature brings minor secondary flow perpendicular to the main flow. This technique can exert additional drag force on particles in addition to the inertial lift forces [45]. For instance, in a curved channel, fluid velocity in the centerline is faster than near the wall regions, which generates a pressure gradient in the radial direction. The inert fluid near the walls recirculates inward and creates 2 symmetric counter-rotating vortices, called Dean vortices [46]. The Dean vortices apply an extra drag force on particles in addition to the inertial lift forces, modifying the original equilibrium positions and accelerating particles to reach the final equilibrium positions [47–49]. For example, introducing curvature in a symmetrically curved channel reduces the equilibrium positions from 4 to 2 [50,51]. To further cut down the equilibrium positions, asymmetrical curvature can be employed, so that a single focusing position can be achieved [51–53]. However, the design of asymmetrically curved channels is rather complex. Moreover, particle focusing position in asymmetrically curved channels is stable and insensitive to particle size, inhibiting the separation of particles and cells based on differential focusing positions [52].

In this work, we proposed a novel way to modify and shrink the focusing positions and enable particle separation based on differential focusing positions by embedding asymmetrical obstacle microstructures. We designed 3 different micro-obstacle structures asymmetrically arranged on the sidewalls of sinusoidal channels. First, we numerically investigated the influence of the asymmetrical obstacles on the flow field of the sinusoidal channels. Next, we experimentally studied particle inertial focusing properties in the channels with different asymmetrical obstacles. We observed the unilateral particle focusing pattern of particles in the channels with concave obstacles and concave–convex obstacles. However, convex obstacles could not alter the inertial focusing pattern evidentially. Finally, we successfully applied the discovered unilateral focusing phenomenon for the separation of a binary polystyrene particle mixture (10 and 15 μm) and isolation of brain cancer cells (U87MG) from white blood cells (WBCs). The results showed that the recovery of cancer cells and WBC rejection rate were as high as 96.4% and 98.81%, respectively. Embedding asymmetric concave micro-obstacles in curvilinear channels may introduce a new way for unilateral inertial focusing and separation.

## Materials and Methods

### Design and fabrication

**Fig. 1.**
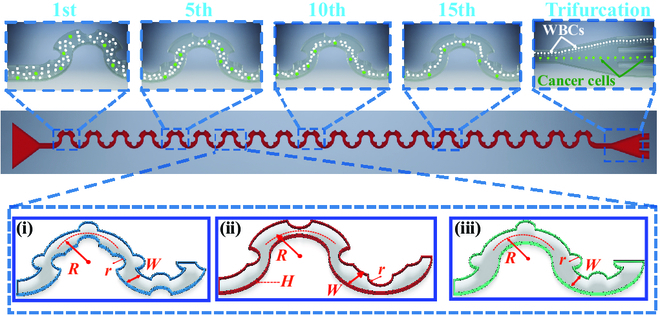
Schematics of particle unilateral focusing and separation in sinusoidal microchannels with asymmetric obstacles: (i) concave and convex obstacles on each respective sidewall (concave–convex obstacle channel), (ii) concave obstacles on one sidewall (one-sided concave obstacle channel), and (iii) convex obstacles on one sidewall (one-sided convex obstacle channel).

We aimed to investigate the effects of asymmetric obstacles on inertial focusing of particles in sinusoidal channels. We patterned periodic semicircular microstructures asymmetrically on the sidewalls of the sinusoidal channel (Fig. 1). We defined that the channels embedded with (a) concave and convex obstacles on each respective sidewall as concave–convex obstacle channel, (b) concave obstacles on one sidewall as one-sided concave obstacle channel, and (c) convex obstacles on one sidewall as one-sided convex obstacle channel (Fig. 1). The radius *r* of the obstacles varies from 50 to 150 μm. The channel width (*W*) and curvature radius (*R*) of the primary sinusoidal channels are 300 and 375 μm, respectively. The sinusoidal channel consisted of 20 repetitive sinusoidal periods so that the channel length is sufficiently long for particles to reach their equilibrium positions. The height of the channel is constant at 50 μm. The sinusoidal channels were fabricated by the standard photolithography and soft lithography [54,55].

### Particle preparation

Spherical polystyrene microparticles of 10 μm (Thermo Fisher Scientific, product no. G1000) and 15 μm (Phosphorex, product no. 1015KB) were suspended in deionized (DI) water, with a particle–weight ratio of around 0.05%. Tween 20 (Sigma-Aldrich, product no. P9416) at a weight ratio of 0.1% was added into the mixture as a surfactant to prevent particle aggregation. To characterize the performance of the device, we used 10- and 15-μm particles in DI water with concentrations of 9.26 × 10^5^ and 2.6 × 10^5^ counts/ml, respectively. The particle concentrations remained consistent for all the experiments in both particle focusing and separation experiments.

### Biological sample preparation

Blood was sourced from the Australian Red Cross Blood Service. The Griffith University Human Research Ethics Committee approved the use of human blood samples with protocol number 2021/598. All experiments were performed in compliance with the relevant laws and institutional guidelines. U87MG human glioblastoma cells were purchased from the American Type Culture Collection (Manassas, VA, USA). The cell culture reagents were purchased from Thermo Fisher Scientific (Waltham, MA, USA). Cells were cultured in T75 flasks in Dulbecco’s modified Eagle’s medium (DMEM) with low glucose, 10% heat-inactivated fetal bovine serum, penicillin (100 U/ml), and streptomycin (100 μg/ml) under humidified atmosphere (37 °C and 5% CO_2_). Cells were then incubated with dihexyloxacarbocyanine iodide (100 ng/ml) overnight under the same conditions as above. The incubated cells were detached with the TrypLE Express Enzyme for 5 min and checked under an Olympus CK40 microscope for detachment. A volume of 4 ml of DMEM was added to the flask. The stained cells were subsequently centrifuged at 700×g for 5 min. The cell pellets were redispersed in a 6-ml DMEM. The stained cells were observed using a microscope to confirm successful staining. To collect WBCs, we used a density gradient medium (Leuko Spin Medium, pluriSelect Life Science UG & Co. KG) to isolate WBCs from the blood sample based on the protocol provided by the company. Briefly, the diluted blood sample was carefully layered on top of the density gradient medium. Then, the sample was centrifuged at 1,000×g for 30 min. The leukocyte cells were washed twice via centrifugation at 300×g with phosphate-buffered saline for 10 min at 4 °C before use. To evaluate the performance of the device on cancer cell separation, the cancer cells were spiked into the WBC sample at a ratio of 1%, where the cancer cell concentration was approximately 5.39 × 10^3^ counts/ml.

### Experimental setup

Microfluidic devices were placed on an inverted microscope stage (Nikon, Eclipse Ts 100). A syringe pump (SHENCHEN ISPLab02) infused particle suspension into the devices at specific flow rates. The testing flow rate ranged from 100 to 2,000 μl/min with an interval of 100 μl/min. A high-speed charge-coupled device camera (Photron, FASTCAM SA3) was mounted on the microscope to capture the trajectories of particles. The typical exposure time was between 2 and 50 μs, and each video comprised at least 300 frames. The open-source software ImageJ (National Institutes of Health) was used to analyze the recorded videos. To quantitatively characterize the particle focusing properties, we used a color-coded map to illustrate the particle distribution along the lateral position. Each color represented the normalized frequency of particles at a specific lateral position [56,57].

The separation tests were carried out to evaluate the performance of the proposed designs. Three criteria, recovery, purity, and rejection ratios were defined to assess separation performance. Purity is the ratio of the number of target particles to the total number of particles at the same outlet/inlet:

Purity=NtargetoutletinletNtotaloutletinlet (1)

Recovery is the ratio of the number of target particles at the specific outlet to the total number of target particles at the inlet:

Recovery=NtargetoutletNtargetinlet=Ntargetoutlet∑Ntargetoutlets (2)

Rejection ratio is the ratio of the number of nontarget/waste cells at the specific outlet to the total number of nontarget/waste cells from all outlets:

Rejection ratio=Nnontargetoutlet∑Nnontargetoutlets (3)

Meanwhile, enrichment is defined as the ratio of the purity of target particles at the outlet (*P*_outlet_) to the purity of target particles at the inlet (*P*_inlet_).

Enrichment=PoutletPinlet=NtargetNtotaloutletNtargetNtotalinlet (4)

## Results and Discussion

### Numerical modeling of fluid flow in channels with asymmetric obstacles

**Fig. 2.**
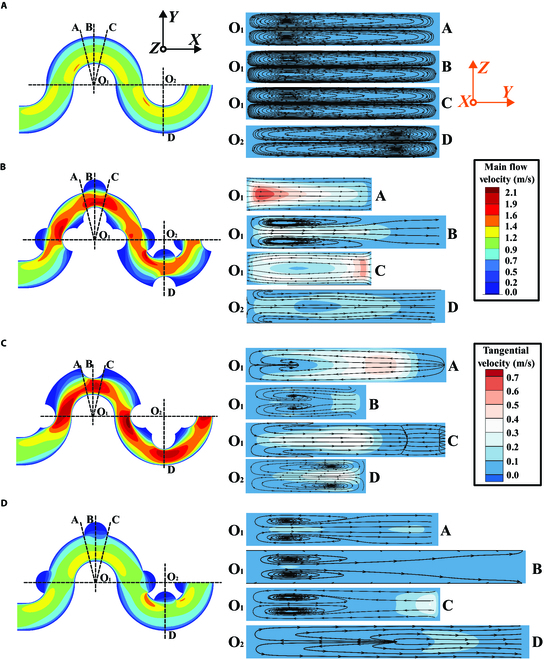
Numerical simulation of the main flow and secondary flow at different cross sections of (A) plain sinusoidal channel with no obstacle, (B) concave–convex obstacle channel, (C) one-sided concave obstacle channel, and (D) one-sided convex obstacle channel. A 2-color legend indicates the velocity magnitude in the primary and secondary flows. The size of the obstacles is 125 μm for all cases. The flow rate is 600 μl/min (*Re* = 56). The angle between O_1_A (or O_1_C) and O_1_B is 14° for all cases. The arrows with lines represent the fluid surface streamlines of secondary flow within the channel cross sections.

We first investigated the fluid flow field in the asymmetrical sinusoidal channels with one-sided concave, one-sided convex, and concave–convex obstacles using numerical simulation with ANSYS 18.1 (Fig. 2). The radius of the obstacles is 125 μm for all cases. The plain channel with no obstacles was simulated at the same flow conditions as a control. The flow rate is 600 μl/min (*Re* = 56). We divided 4 cut planes of OA–OD along the sinusoidal shape to exhibit the secondary flow in the cross section. The selection of the cross sections was based on the aim to comprehensively capture the salient characteristics of each obstacle. Specifically, the planes that traverse the corners of these features were chosen to investigate the maximum velocity fluctuations occurring before, during, and after the passage of the obstacles. In each cross section, the arrows with lines represent the fluid surface streamlines of secondary flow.

**Fig. 3.**
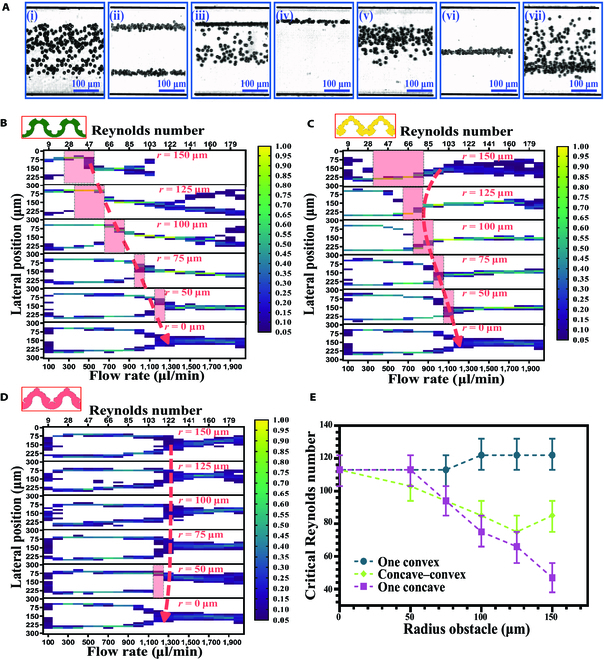
Inertial focusing in sinusoidal channels with various asymmetric obstacles. (A) Bright-field images illustrate 7 typical particle focusing behaviors in the channels with asymmetric obstacles: (i) random distribution, (ii) 2-position focusing, (iii) transition to the unilateral position focusing, (iv) unilateral position focusing, (v) transition to the single middle position focusing, (vi) single middle position focusing, and (vii) defocusing. (B to D) The influence of obstacle radius on the inertial focusing of particles at concave–convex obstacle channel, one-sided concave obstacle channel, and one-sided convex obstacle channel. The color legend indicates the normalized frequency of particles at a specific lateral position of the channel. (E) The critical Reynolds number in different obstacle channels with different obstacle radii (from 50 to 150 μm). The critical Reynolds number (*Re*_C_) is the minimum value where unilateral focusing streaks convert into a single central focusing pattern. We employed error bars to represent the error to determine the value of the critical Reynolds number. The spherical polystyrene particles are 10 μm in diameter.

Similar to obstacles that are symmetrically patterned in sinusoidal channels [56], the asymmetrical obstacle pattern can also boost the Dean flow velocity (tangential velocity) (Fig. 2). This enhanced secondary flow accelerates particle movement to the final equilibrium points. In addition, the location of the circulating vortices has shifted in the cross sections. For example, in a channel with no obstacles, 2 symmetrical vortices with circulating streamlines occupy the whole cross sections. However, in a channel with asymmetrical obstacles, the vortices shift to the inner wall regions, affecting the final equilibrium positions of particles in the cross sections. The particle shifting behaviors will be more pronounced after repetitive alternation in series, finally resulting in unilateral focusing trajectories of particles, as depicted in subfigure (iv) of Fig. 3A.

Furthermore, the sudden contraction in a channel with asymmetrical obstacles generates a net deformation of the fluid streams. We can observe the phenomenon of stream fractionation because of the concave obstacle, especially in the region with the sharp edge (the blue regions before and after the obstacle structure). The flow in the cross sections is twisted irreversibly and loses symmetry before and after the concave obstacle. The opposite direction of secondary flow before (cross section O_1_A) and after (cross section O_1_C) obstacle regions can be observed (Fig. 2B to D). This was also reported in the straight and spiral channels with obstacles [45,58,59].

In summary, the asymmetrical obstacle microstructures boost the main flow velocity, modifying the magnitude and direction of secondary flow in cross sections. This combination will break the original balance between inertial lift and Dean drag forces, destabilizing the symmetric focusing behaviors [39]. Reducing the symmetry of the inertial focusing in sinusoidal channels is expected to result in new unilateral focusing trajectories. In the following sections, we will experimentally verify this hypothesis.

### Effects of 3 asymmetric obstacle patterns

To investigate the effect of asymmetrical obstacle structures on particle inertial focusing, we experimentally tested the trajectories of 10-μm particles in the channel with 3 different asymmetric obstacle patterns. In this study, the obstacles had 5 different radii (50, 75, 100, 125, and 150 μm) in these 3 patterns. The inlet flow rate of particle suspension ranged from 100 to 2,000 μl/min. Figure 3B to D shows the normalized lateral distribution of particles under different flow rates (Reynolds number).

Our previous study examined how symmetrical obstacle patterns placed on 2 sidewalls of sinusoidal channels affect inertial focusing [56]. The study found that these structures influenced the strength of Dean flow and the flow rate for particle focusing and separation. Moreover, shape of obstacles may minorly affect flow rates for inertial separation, but it does not alter the focusing patterns [60]. However, the overall shape of the focusing patterns remained similar to those observed in sinusoidal channels without obstacles. In contrast, compared with the symmetrical design, the main difference is that unique unilateral focusing pattern regions exist between the 2-sided focusing and single central focusing for the asymmetric concave obstacle channel (Fig. 3A and B). In the first stage, the particles were focused near 2 sidewalls at a relatively low Reynolds number, where the Dean flow could not compete with the original inertial equilibrium positions (subfigure (ii) of Fig. 3A). Next, particles from the opposite side of concave obstacles were destabilized and gradually merged into particle focusing stream on the other side when the Reynolds number increased, eventually developing into a unilateral focusing stream (subfigures (iii) and (iv) of Fig. 3A). This phenomenon can be explained by the fact that the asymmetrical obstacles cause fluid deformation and modify secondary flow to break the original equilibrium position. The original symmetrical focusing is destabilized, and the number of particle focusing locations is reduced, resulting in unilateral focusing. Further enhancing the flow rate, unilateral focusing shifted gradually toward the channel center and then formed a single central focusing (subfigures (v) and (vi) of Fig. 3A). Above a certain threshold, particle defocusing arose because of the dominant mixing effect of secondary flows (subfigure (vii) of Fig. 3A).

In addition, the region of the unilateral focusing (the red shade region) was stretched when increasing the obstacle size (Fig. 3B). The larger the obstacle size, the lower the initiating flow rate of unilateral focusing. The underlying mechanism is that the concave obstacle structure can boost the Dean vortex by increasing the obstacle size [56]. Therefore, the enhanced Dean flow can enable the unilateral inertial focusing on a lower Reynolds number. Meanwhile, the concave–convex obstacle channel has similar focusing patterns as the one-sided concave obstacle channel (Fig. 3C). However, note that the starting Reynolds number of the unilateral focusing is generally higher compared to the concave obstacle channel with the same obstacle size. This is because the main velocity of the concave–convex obstacle channel is smaller than the one-concave obstacle channels, leading to a less-significant local secondary flow to speed up the particle movement. However, the apparent change in particle focusing pattern was hardly observed in the one-sided convex obstacle channels, and the particle focusing behavior was similar to the no-obstacle channel (*r* = 0).

Furthermore, we utilized the critical Reynolds number (*Re*_C_) to quantitatively evaluate the effects of obstacle size on particle focusing. The critical Reynolds number represents the transition from sided (unilateral or 2-sided) focusing move into single-central focusing, which highly relates to the obstacle pattern and size (Fig. 3E). For both one-sided concave and concave–convex obstacle channels, the larger the obstacle size, the smaller the critical Reynolds number. This means that a smaller Reynolds number can achieve the single central focusing pattern with a larger obstacle. In contrast, the obstacle size in the one-sided convex obstacle channel had a negligible influence on particle focusing (Fig. 3E).

### Unilateral inertial focusing and separation of polystyrene beads

**Fig. 4.**
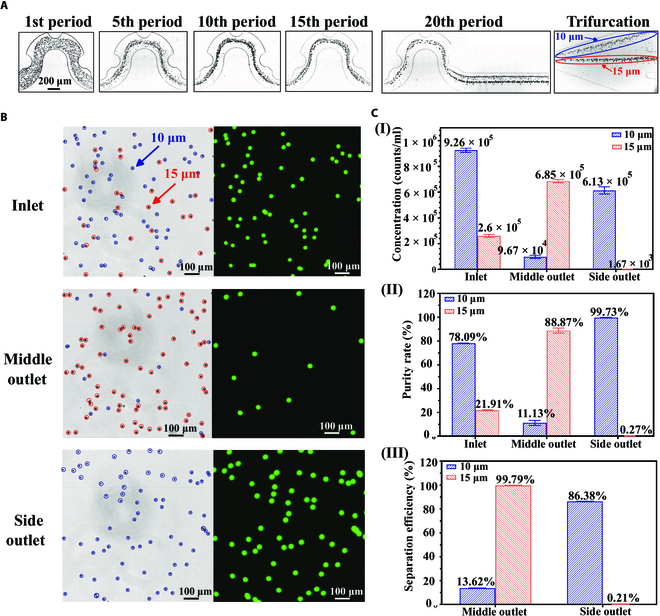
Separation of particles based on different unilateral focusing positions of 10- and 15-μm particles. (A) The bright-field images show the distribution of the particles at 5 specific periodic locations and trifurcation regions of a one-sided concave obstacle channel. The radius of obstacles is 125 μm. (B) The bright-field and fluorescent images of particle mixture under a hemocytometer before and after separation. Ten-micrometer particles have green fluorescence, while 15-μm particles have no fluorescence. (C) The concentration, purity, and separation efficiency of particles after one single processing at a flow rate of 610 μl/min.

After discovering that asymmetrically patterned obstacles can break the symmetricity of inertial focusing, we explored the application of unilateral focusing for particle separation. We observed the differential inertial focusing properties of 10- and 15-μm particles in channels with asymmetrical obstacles (Fig. S1 and Movie S1). Furthermore, we separated particles on the basis of the unique unilateral inertial focusing behavior of 10- and 15-μm particles in the one-sided concave obstacle channel (*r* = 125 μm). The mixture consisted of 10- and 15-μm particles with a ratio of 3.5:1, and the mixture was injected into the device at a flow rate of 610 μl/min. The bright-field images of particle distribution at 6 typical positions (1st, 5th, 10th, 15th, 20th, and trifurcation) of the channel showed the distinct particle migration behavior of 10- and 15-μm particles (Fig. 4A). The boosted Dean secondary flow adjusted the particle trajectories, and the 15-μm particles experienced a more significant effect of Dean flow, taking the lead in merging at channel center, while the 10-μm particles were at the unilateral focusing position. Finally, they were separated at the trifurcation region.

Subsequently, we collected the separated samples from outlets and counted the number of particles with a hemocytometer. We could see that most 10-μm particles were removed from the mixture and separated into the side outlet. At the same time, 15-μm particles were concentrated significantly at the middle outlet (Fig. 4B). We also quantitatively characterized the separation performance (Fig. 4C). The separation efficiency was as high as 99.79%, and the purity was 88.87% for 15-μm particles. Meanwhile, the separation efficiency for the 10-μm particles was 86.38%, with a high purity of 99.73% at the side outlet after one single process.

### Separation of cancer cells from WBCs by differential unilateral focusing

The circulating tumor cells (CTCs) in the peripheral blood of patients with cancer are emerging as a promising biomarker for cancer diagnosis, prognosis, and therapy monitoring [61]. Isolation of CTCs from the whole blood is indispensable for CTC-based liquid biopsy. In the whole blood, red blood cells can be depleted by cell lysis buffer and centrifugation. Thus, isolation of CTCs from WBCs is the most critical process. As a proof of concept, we applied the one-sided concave obstacle channel device (*r* = 125 μm) to separate cancer cells from WBCs based on the differential unilateral focusing positions. We first separated WBCs from whole blood using density gradient centrifugation and then spiked the fluorescently labeled brain cancer cells (U89MG) into WBCs suspension. The spiking ratio between WBCs and cancer cells was around 100:1. The size of cancer cells was from 14 to 20 μm, with most in the range of 16 to 18 μm, relatively larger than most WBCs (8 to 12 μm) [62].

**Fig. 5.**
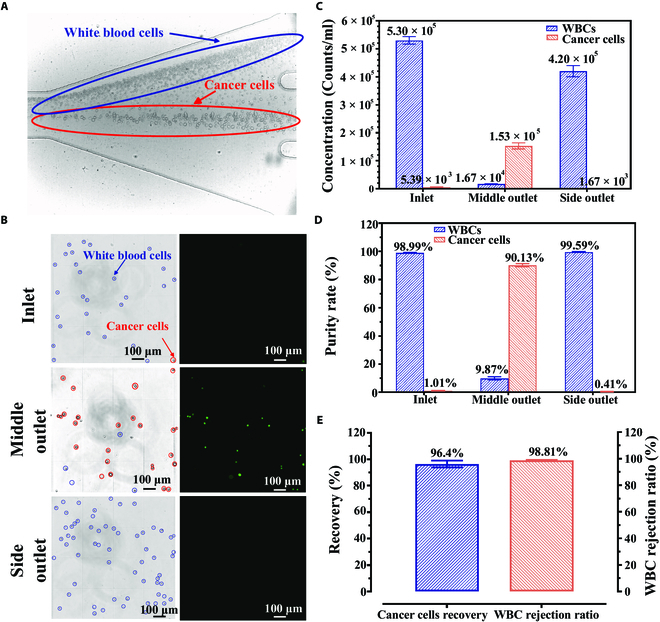
Separation of white blood cells (WBCs) and cancer cells based on differential unilateral focusing in a one-sided concave obstacle channel. (A) Trajectories of WBCs and cancer cells at the outlet trifurcation area. (B) Microscopic images of blood cell samples at the inlet and outlets under a hemocytometer. The cancer cells are stained with green fluorescence. (C) Concentration and (D) purity of cancer cells and WBCs before and after separation after one single processing through the one-sided concave obstacle channel. (E) The recovery of the cancer cells and rejection ratio of WBCs after one single processing. The spiking ratio of cancer cells to WBCs is approximately 1:100.

On the basis of the unilateral focusing pattern, WBCs were continuously depleted from the one-sided outlet, and most cancer cells were isolated and purified from the middle outlet (Fig. 5A and Movie S2). Furthermore, bright-field and fluorescent microscopic images illustrated the ratios of WBCs and cancer cells in the sample before and after separation (Fig. 5B). Before separation, WBCs dominated the sample mixture, and the cancer cells were hardly detected. However, after single processing via the one-sided concave obstacle channel, cancer cells were highly purified and concentrated at the middle outlet, with a majority of WBCs extracted from other outlets. After single processing, the purity of the cancer cells dramatically increased from 1.01% to 90.13%, with an 89.24-fold enrichment (Fig. 5C and D). Moreover, the recovery of cancer cells and WBC rejection rate reached 96.4% and 98.81%, respectively (Fig. 5E).

In summary, incorporation of asymmetric obstacles in sinusoidal channels can significantly decrease the number of focused particles, which enables unilateral particle focusing. Capitalizing on this unique property, we have demonstrated the feasibility of using unilateral focusing for size-based particle and cell separation. This approach holds significant promise for other applications, such as plasma extraction, where unilateral focusing may facilitate the separation of cells and plasma into separate outlets. Moreover, the reduced number of focusing streams along the channel sidewall could enable easy integration of additional working units, potentially enhancing the overall separation performance.

## Conclusion

We introduced a new method to adjust and reduce inertial focusing positions by embedding asymmetrical obstacles in the channels. Three asymmetrical obstacle patterns, such as one-sided concave, one-sided convex, and concave–convex obstacle patterns, were embedded in the symmetric sinusoidal channels. We observed the unique unilateral focusing pattern near the sidewalls in the one-sided concave and concave–convex obstacle channels, while insignificant in the one-sided convex obstacle channels. In addition, as the size of the obstacles increased, the starting flow rate of the unilateral focusing pattern reduced, and the flow rate region for this focusing mode was stretched out. Furthermore, the position of unilateral focusing was sensitive to particle size. Therefore, we applied the differential unilateral focusing on separating 10- and 15-μm particles and isolating cancer cells (U87MG) from WBCs. The purity of the cancer cells was significantly enhanced from 1.01% to 90.13%, with an 89.24-fold enrichment after single processing. The cancer cell recovery was as high as 96.4%, with a WBC rejection ratio of 98.81%. Embedding asymmetric concave micro-obstacles in curved channels offers a new strategy to achieve unilateral inertial focusing and separation.

## Data Availability

Data are available upon reasonable request.
