# Supplementary material for: Asymmetrical Obstacles Enable Unilateral Inertial Focusing and Separation in Sinusoidal Microchannel
Source: Cyborg Bionic Syst. 2023 Jun 19;4:0036. doi: 10.34133/cbsystems.0036 (PMC10278993; doi:10.34133/cbsystems.0036)
Supplement: Supplementary 1 — Fig. S1 [file cbsystems.0036.f1.docx]

***Supplymentary Information***

**Asymmetrical obstacles enable unilateral inertial focusing and separation in sinusoidal microchannels**

Haotian Cha^a^, Yuchen Dai^a^, Helena H.W.B. Hansen^a^, Lingxi Ouyang^a^, Xiangxun Chen^a^, Xiaoyue Kang^b^, Hongjie An^a^, Hang Thu Ta^a,c^, Nam-Trung Nguyen^*a^, Jun Zhang^*a^

^a^ Queensland Micro- and Nanotechnology Centre, Griffith University, Nathan, Queensland 4111, Australia. E-mail: jun.zhang@griffith.edu.au; nam-trung.nguyen@griffith.edu.au

^b^ School of Engineering, University of Tasmania, Churchill Avenue, Tasmania 7005, Australia

^c^ Bioscience Discipline, School of Environment and Science, Griffith University, Nathan, Queensland 4111, Australia

**Table of contents**

S-1. The inertial particle-focusing trajectories for 10 and 15 µm particles in asymmetrical obstacle patterns.

Video S1. Unilateral inertial focusing and separation of 10 and 15 µm polystyrene particles in one-sided concave obstacle channel.

Video S2. Unilateral separation of cancer cells from white blood cells in one-sided concave obstacle channel.

# **Figure S1. The inertial particle-focusing trajectories for 10 and 15 µm particles in asymmetrical obstacle patterns.**

We first analyze the effect of particle size on the asymmetrical obstacle patterns, Fig. S1. The red-shaded part presents the duration of the unilateral focusing pattern. The blue dotted line shows the potential separation region from the unilateral side and the middle regions. Among all three-obstacle patterns, the starting point of the one-side focusing pattern occurs earlier when focusing on the larger particles. This phenomenon can be explained by the magnitude of the Dean drag force, which positively correlates to the size of the particle and can advance the unilateral focusing pattern at a relatively lower Reynolds number. It is noted that this phenomenon is more obvious in the one-concave obstacle structure.


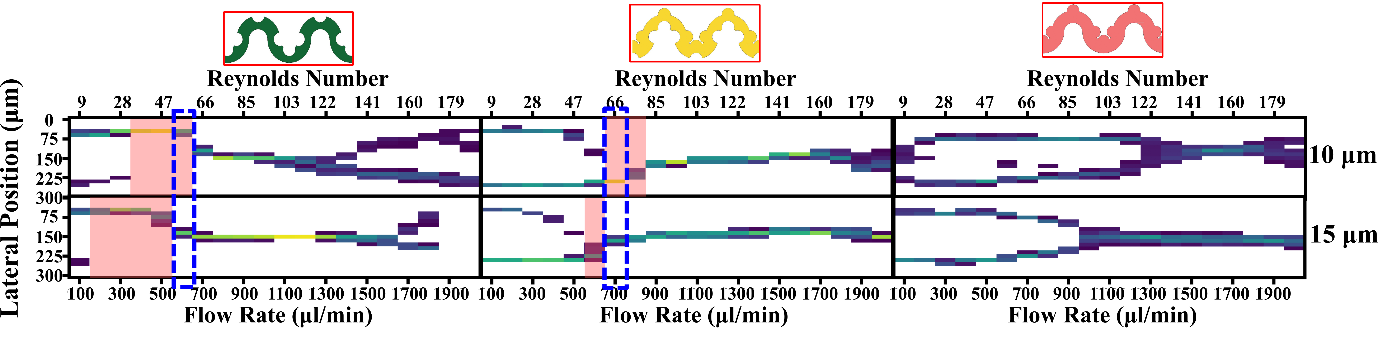


Fig. S1. The colour map of 10 µm and 15 µm particles under various flow rates among three asymmetrical designs. The red shade part presents the duration of the unilateral focusing pattern. The blue dotted line shows the potential unilateral separation region. The obstacle size was 125 µm for all cases.

**Video S1. Unilateral inertial focusing and separation of 10 and 15 µm polystyrene particles in one-sided concave obstacle channel.**

**Video S2. Unilateral separation of cancer cells from white blood cells in one-sided concave obstacle channel**.
